# Endonasal access to the lateral poststyloid space: Far lateral extension of an endoscopic endonasal corridor

**DOI:** 10.1002/hed.27135

**Published:** 2022-06-29

**Authors:** Lifeng Li, Nyall R. London, Leslie R. Kim, Daniel M. Prevedello, Ricardo L. Carrau

**Affiliations:** ^1^ Department of Otolaryngology – Head & Neck Surgery, Beijing Tongren Hospital Capital Medical University Beijing China; ^2^ Department of Otolaryngology – Head & Neck Surgery The James Cancer Hospital at the Wexner Medical Center of The Ohio State University Columbus Ohio; ^3^ Department of Otolaryngology – Head & Neck Surgery Johns Hopkins School of Medicine Baltimore Maryland USA; ^4^ Department of Neurological Surgery The James Cancer Hospital at the Wexner Medical Center of The Ohio State University Columbus Ohio

**Keywords:** endonasal, facial nerve, lateral, poststyloid space, styloid process

## Abstract

The styloid process constitutes the posterolateral boundary for an endonasal exposure of the infratemporal fossa. This study aims to explore the feasibility of a far‐lateral extension to the lateral poststyloid space via an endonasal corridor. An endonasal dissection was performed on six cadaveric specimens (12 sides). Following an endoscopic endonasal access to the parapharyngeal space, the styloid process and the tympanic portion of the temporal bone were removed to reveal the jugular bulb and the extratemporal facial nerve. Distances from the anterior nasal spine to the relevant landmarks were measured using a surgical navigation device. Through an endonasal corridor, only the anteroinferior aspect of the jugular bulb was exposed. Conversely, the extratemporal facial nerve could be sufficiently exposed, and the deep temporal nerve could be transposed to the stylomastoid foramen. The average horizontal distances from the nasal spine to the posterior tract of V_3_, styloid process, and facial nerve were 79.33 ± 3.41, 97.10 ± 4.74, and 104.77 ± 4.42 mm, respectively. Access to the lateral poststyloid space via an endonasal corridor is feasible, potentially providing an alternative approach to address select lesions extending to this region. The deep temporal nerve has a similar diameter to that of the facial nerve; thus, providing potential reinnervation of the facial nerve.

## 1 INTRODUCTION

Expanded endonasal approaches (EEA) to the skull base have undergone sustained advancements, providing the advantage of avoiding brain retraction.^1^, ^2^, ^3^ The cavernous sinus, anteromedial aspect of the Meckel's cave, petrosal apex, petroclival region, and ventral aspect of the brainstem can all be accessed directly through an EEA corridor.^4^, ^5^, ^6^ Furthermore, an EEA can also avoid external incisions and are associated with a shortened stay in the hospital, superior postoperative quality of life, and satisfactory surgical outcomes.^7^, ^8^

Prior to the advent of EEA, the preauricular subtemporal or infratemporal approach (type B, C) was commonly chosen to address lesions arising in the infratemporal fossa (ITF) and upper parapharyngeal space (UPPS).^9^, ^10^ Drawbacks associated with this and other open approaches such as the facial incision, need for orbital and zygomatic osteotomies, potential of damage to the facial nerve, temporalis muscle and branches of mandibular nerve (V_3_),^11^ which have triggered the continual emergence of minimally invasive techniques (i.e., endonasal transpterygoid approach) for dissection and extirpation of tumors arising in ITF and UPPS.^12^, ^13^

Endoscopic endonasal approaches accessing the UPPS have been well described.^4^, ^14^, ^15^, ^16^, ^17^ During these approaches, it is critical to avoid inadvertent injury to structures posterior to the styloid process (retro‐styloid compartment) including the parapharyngeal internal carotid artery (pICA), internal jugular vein, and lower cranial nerves (CN IX to XII).^18^ Therefore, the styloid process is considered as the posterolateral boundary of the dissection. Nonetheless, with advances in instrumentation and surgical techniques, utilization of an EEA has potentiated extending the access in a posterolateral trajectory toward the jugular bulb and facial nerve.

Studies regarding the styloid process and the adjacent anatomical relationships from an endoscopic endonasal perspective are sparse. Therefore, this study aims to assess the feasibility of accessing the area posterolateral to the styloid process through an endoscopic endonasal approach and test the hypothesis that anatomic structures of this region including the facial nerve and the anteroinferior aspect of the jugular bulb could be adequately reached via an EEA corridor.

## 2 MATERIALS AND METHODS

### 2.1 Cadaveric dissection

An endoscopic Denker's approach was performed to access the posterolateral‐styloid space in six adult cadaveric specimens (12 sides). These dissections were performed at the Anatomy Laboratory Toward Visuospatial Surgical Innovations in Otolaryngology and Neurosurgery (ALT‐VISION) at the Wexner Medical Center of The Ohio State University. ALT‐VISION and all coauthors involved in the dissections were certified by local regulatory agencies dealing with the use of human tissues and cadaveric studies. The common carotid and vertebral arteries as well as the internal jugular veins were identified and injected with red and blue silicone dyes, respectively. All specimens were preserved in 70% alcohol. High‐resolution CT scans was performed, and the images were imported to a Stryker navigational system (Kalamazoo, MI).

We used 0°, 30°, and 45° lenses (4‐mm diameter, 18‐cm length) coupled to a high‐definition camera and monitor (Karl Storz Endoscopy, Tuttlingen, Germany) to provide visualization. An AIDA system (Karl Storz Endoscopy, Tuttlingen, Germany) was used to record and save images (TIF format) and videos (MPEG format). Both still photographs and videos were obtained to define and document the anatomic relationships of the endoscopic anatomy and correlate dissections with the multiplanar CT views provided by the image guidance system. We used high‐speed drill (Stryker Co., Kalamazoo, MI) with straight hand‐piece and 3–4 mm coarse diamond burrs.

### 2.2 Radiological measurements

CT scans of the six cadaveric specimens comprising axial, coronal, and sagittal images in Digital Imaging and Communications in Medicine (DICOM) format were obtained and imported into a navigation system (Stryker Navigation; Kalamazoo, Michigan). Distances from the anterior nasal spine to the posterior trunk of V_3_, styloid process, and the facial nerve at the level of the nasal floor were recorded and measured using the navigation system in the axial plane. The results were recorded as mean ± standard deviation (SD).

### 2.3 Three‐dimensional reconstruction

Image data incorporating the coronal, axial, and sagittal images of both the CT and MRI were obtained in the format of Digital Imaging and Communications in Medicine. This was imported into an IVSP Image software (IVSPlan, Beijing, China) for further segmentation and reconstruction. On each image plane, the arteries, veins, tumors, and bony facets (maxillary, mandibular, and temporal bone) were segmented separately.

Following image segmentation, a 3D model including the tumor, great vessels (common carotid artery, ICA, and the internal jugular vein), and the skull base was reconstructed with each portion created separately and then assembled together.

## 3 RESULTS

**FIGURE 1:**
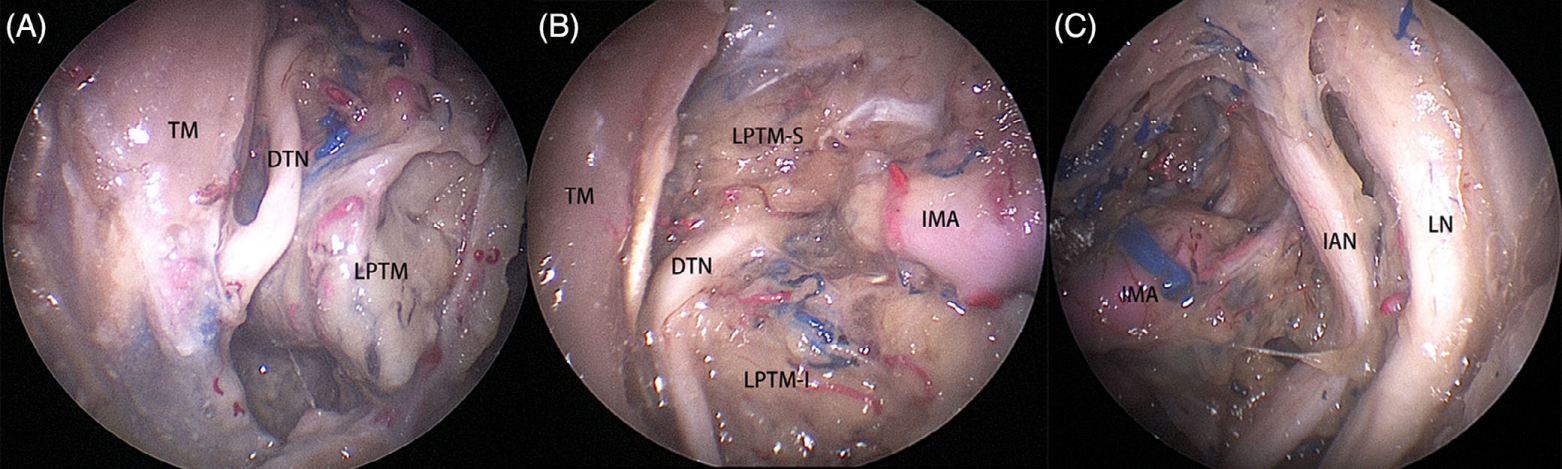
Structures on right side. (A) The deep temporal nerve (DTN); (B) the DTN constituted the separation of the superior (LPTM‐S) and inferior (LPTM‐I) heads of lateral pterygoid muscle; (C) the posterior tract of V_3_. IAN, inferior alveolar nerve; IMA, internal maxillary artery; LN, lingual nerve [Color figure can be viewed at wileyonlinelibrary.com]

Technical nuances for an endoscopic Denker's approach have been detailed in a previous study.^19^ The posterolateral wall of maxillary sinus and the periosteum were removed to reveal the anterior aspect of the ITF. Branches of the internal maxillary artery were sacrificed as necessary to enhance exposure. The deep temporal nerve ran consistently along the medial border of the temporalis muscle in all 12 sides (Figure 1A). When tracing the deep temporal nerve proximally toward the foramen ovale, we identified that this branch pierces between the superior and inferior heads of the lateral pterygoid muscle (Figure 1B). The lateral pterygoid muscle was subsequently transected and elevated from the lateral pterygoid plate to reveal the posterior trunk of V_3_ (Figure 1C).

**FIGURE 2:**
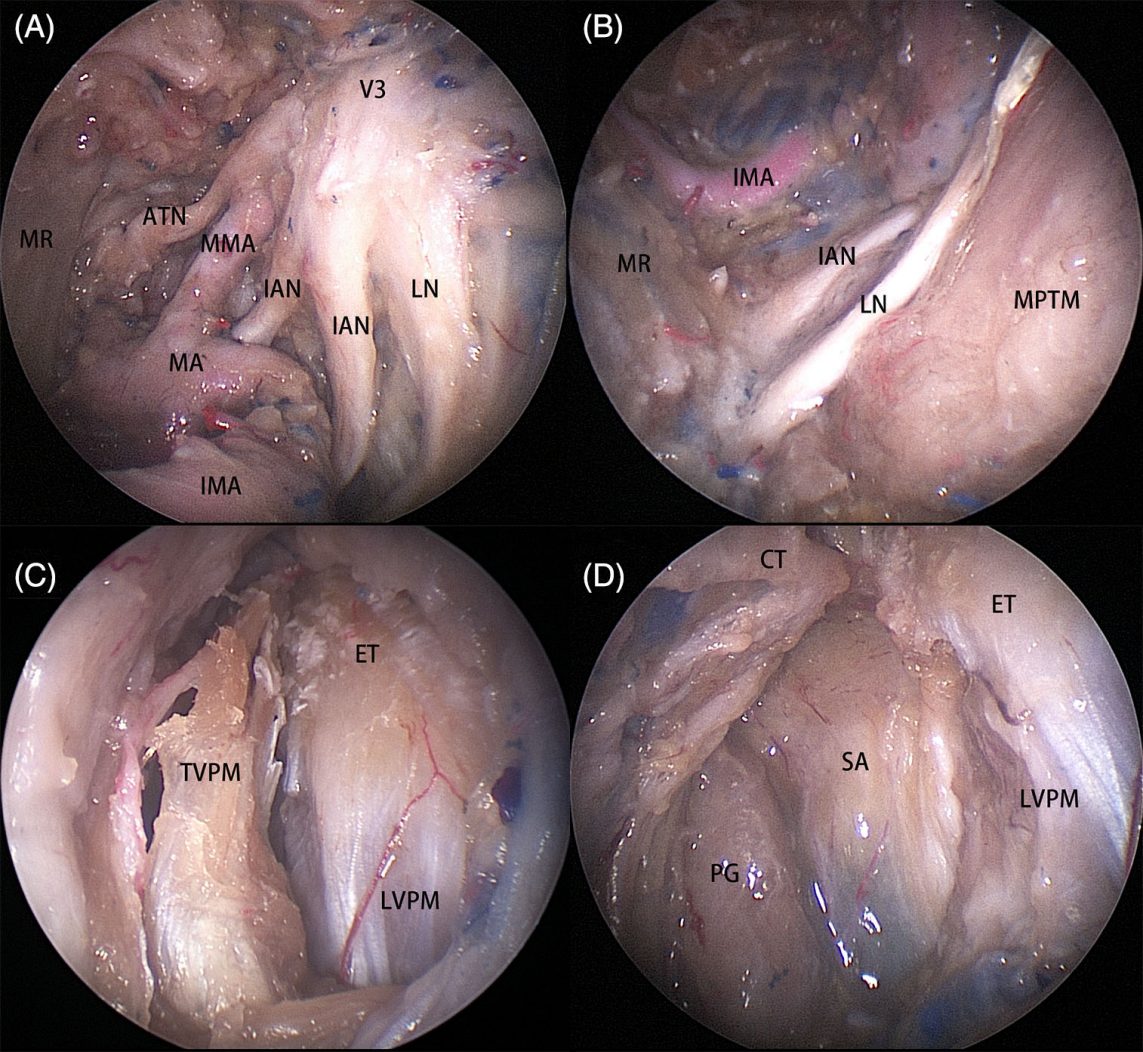
Structures on right side. (A) The branches of posterior trunk of V_3_ includes the lingual nerve (LN), inferior alveolar nerve (IAN), and auriculotemporal nerve (ATN); (B) the LN and IAN travel to the inferolateral aspect; (C) the tensor veli palatini muscle (TVPM) was incised to expose the Eustachian tube (ET) and levator veli palatini muscle (LVPM); (D) the deep lobe of parotid gland (PG) and styloid aponeurosis (SA). CT, chorda tympani; IMA, internal maxillary artery; MMA, middle meningeal artery; MPTM, medial pterygoid muscle; MR, mandible ramus [Color figure can be viewed at wileyonlinelibrary.com]

The posterior trunk of V_3_ including the lingual nerve, inferior alveolar nerve, and auriculotemporal nerve travels parallel to the middle meningeal artery (Figure 2A). The lingual nerve and inferior alveolar nerve ran on the upper surface of medial pterygoid muscle toward an inferolateral direction (Figure 2B). After lateral displacement of the posterior trunk of V_3_, the fat in the prestyloid UPPS was identified. The tensor veli palatini muscle was then transected to reveal the cartilaginous Eustachian tube and the levator veli palatini muscle (Figure 2C). The fat was carefully removed to expose the deep lobe of the parotid gland and the stylopharyngeal aponeurosis (Figure 2D).

**FIGURE 3:**
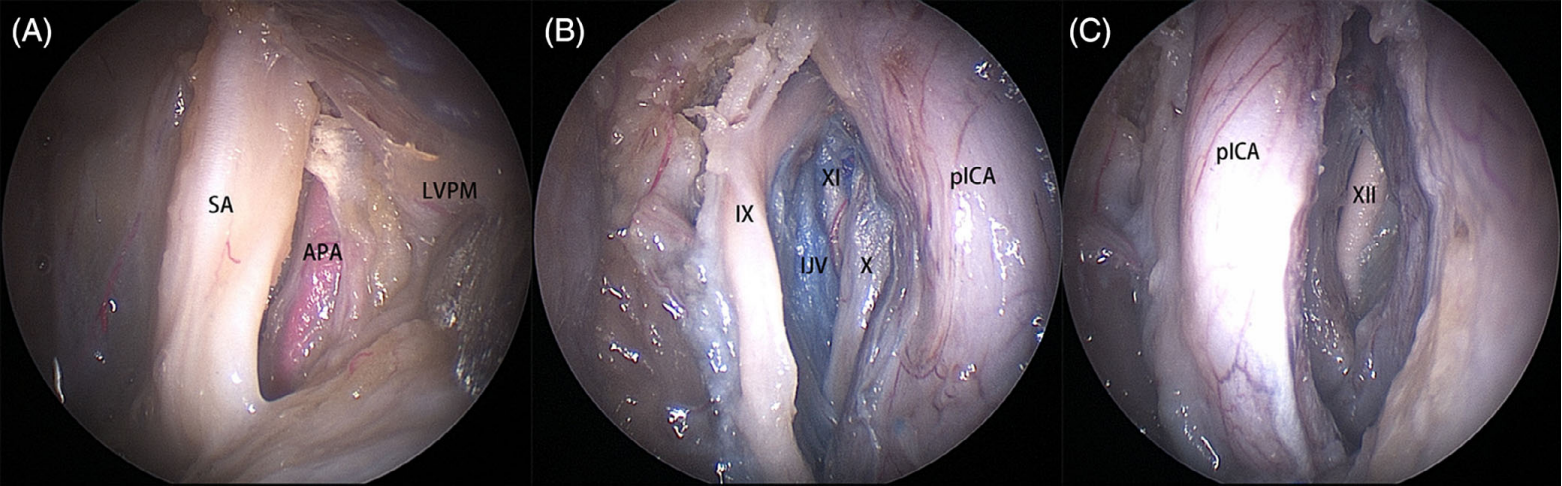
Structures on right side. (A) At the lateral border of levator veli palatini muscle (LVPM), the stylopharyngeal aponeurosis (SA) was incised and the ascending pharyngeal artery (APA) was identified; (B) the cranial nerve IX, X, XI and internal jugular vein (IJV); (C) cranial nerve XII [Color figure can be viewed at wileyonlinelibrary.com]

The stylopharyngeal aponeurosis was incised at the lateral border of levator veli palatini muscle, and the ascending pharyngeal artery was subsequently encountered (Figure 3A). The stylopharyngeal aponeurosis was then peeled from a medial to lateral fashion, and the pICA, CN IX, X, XI and the internal jugular vein were subsequently exposed (Figure 3B). The hypoglossal nerve (CN XII) could be identified after anterolateral displacement of the pICA (Figure 3C).

**FIGURE 4:**
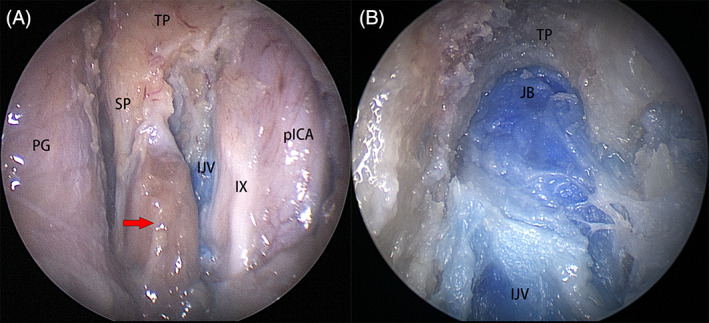
Structures on right side. (A) The tympanic portion (TP) of the temporal bone was apparent, the styloid process (SP) and the attached muscle (arrow) located at its lower aspect; (B) the anteroinferior portion of jugular bulb (JB). IJV, internal jugular vein; PG, parotid gland [Color figure can be viewed at wileyonlinelibrary.com]

In 5/12 sides (41.67%), the tympanic portion of the temporal bone was prominent, and the styloid process was detected at its lower aspect (Figure 4A). The tympanic portion of the temporal bone was removed with a drill to expose the upper segment of the internal jugular vein and the jugular bulb (Figure 4B).

**FIGURE 5:**
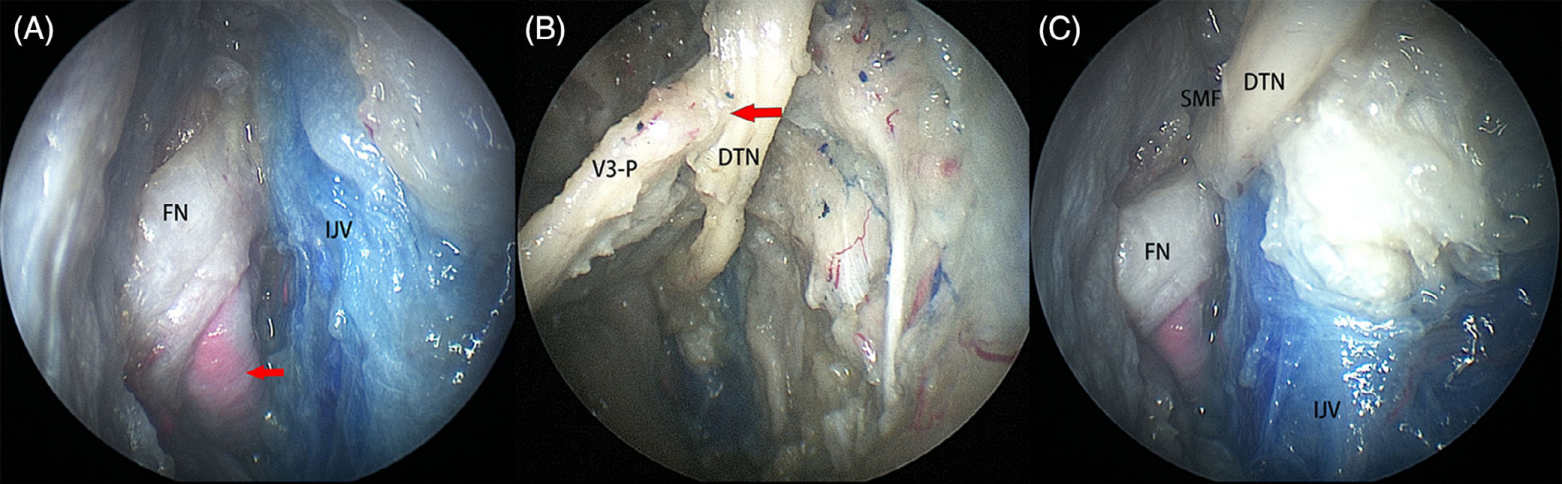
Structures on right side. (A) The facial nerve (FN) and the accompanying vessel (arrow) in the lateral poststyloid space; (B) the deep temporal nerve (DTN) was transected and transposed under (arrow) the posterior trunk of V_3_ (V_3_‐P) to the lateral poststyloid space; (C) the DTN was transposed to level of stylomastoid foramen (SMF) and the potential for anastomosis and reanimation with the facial nerve (FN) was assessed. IJV, internal jugular vein [Color figure can be viewed at wileyonlinelibrary.com]

The muscles and ligaments attached to the styloid process were sharply transected and the styloid process was removed to further expose the anterior aspect of the internal jugular vein, the facial nerve, and its accompanying vessel (Figure 5A).

At the end of the procedure, the deep temporal nerve was transected at its distal end and transposed (Figure 5B) posterolaterally to the level of the stylomastoid foramen to evaluate the possibility of anastomosis with the distal facial nerve, potentially serving as an alternative means for facial reanimation (Figure 5C). These two nerves are almost identical in diameter, and the length of the transposed deep temporal nerve was adequate to reach the stylomastoid foramen and the extratemporal segment of the facial nerve.

The average horizontal distances from the anterior nasal spine to the posterior trunk of V_3_, styloid process, and facial nerve was 79.33 ± 3.41, 97.10 ± 4.74, and 104.77 ± 4.42 mm, respectively (Table 1).

**TABLE 1:** The measurement of distances (at level of nasal floor, mm) from anterior nasal spine to posterior trunk of V_3_, styloid process, and the main trunk of facial nerve

Side	Nasal spine to V_3_	Nasal spine to styloid process	Nasal spine to facial nerve
1	83.51	103.12	111.91
2	82.7	104.05	111.42
3	76.58	91.16	99.87
4	77.23	93.08	101.42
5	80.66	100.72	109.13
6	84.28	102.24	109.45
7	75.35	99.84	103.47
8	77.18	95.32	102.86
9	73.95	90.16	99.98
10	77.89	94.32	103.42
11	82.26	94.95	102.06
12	80.32	96.28	102.23
Mean ± SD	79.33 ± 3.41	97.10 ± 4.74	104.77 ± 4.42

**FIGURE 6:**
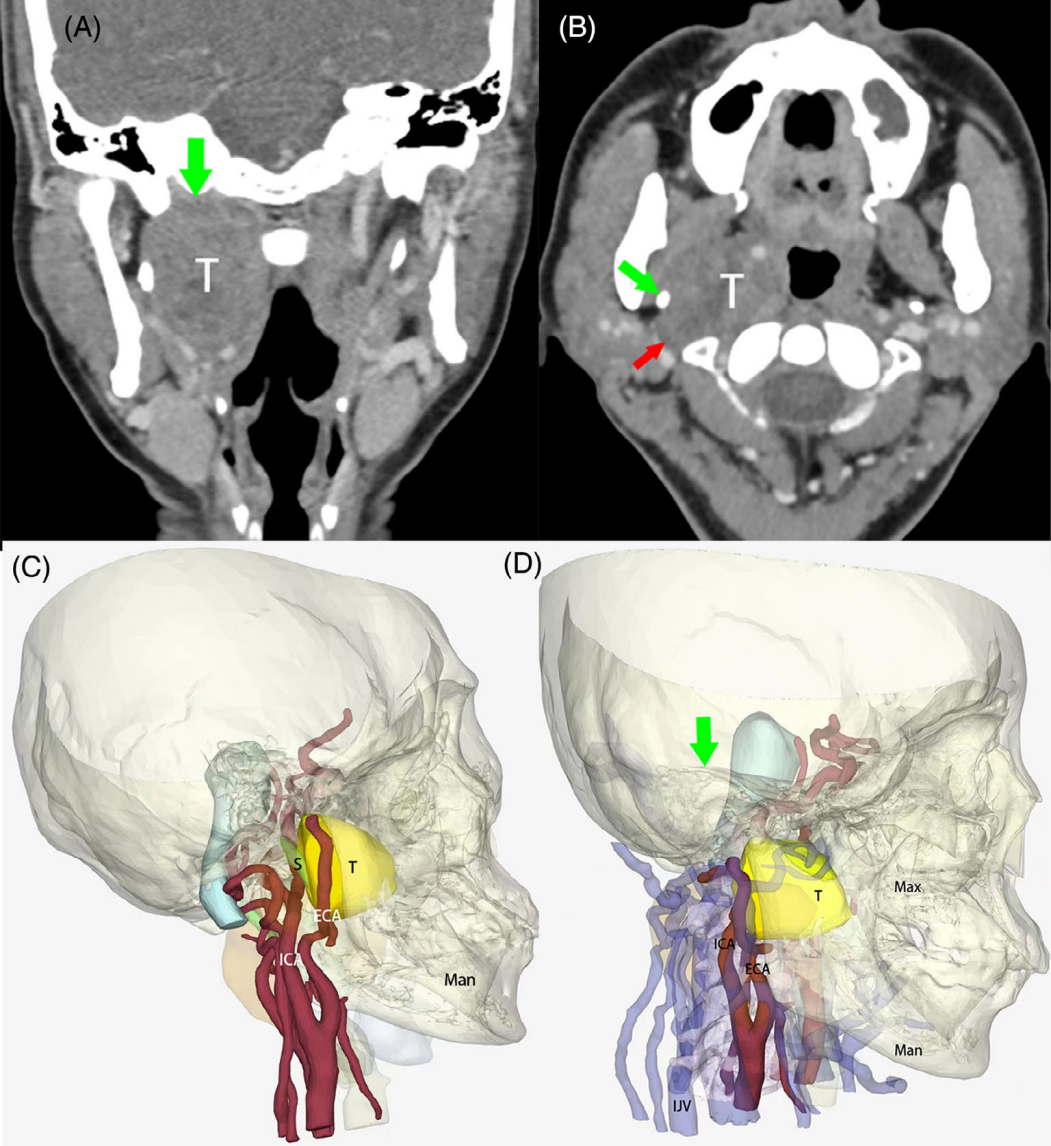
(A) A tumor (T) in the parapharyngeal space was observed, green arrow: middle cranial fossa. (B) A small portion of the tumor (red arrow) was detected to extend posterolateral to the styloid process (green arrow). (C, D) A 3D reconstruction to illustrate the relationship between the tumor (T), the styloid process and the adjacent was performed. ECA, external carotid artery; green arrow, middle cranial fossa; ICA, internal carotid artery; IJV, internal jugular vein; Man, mandibular; Max, maxillary; S, styloid process [Color figure can be viewed at wileyonlinelibrary.com]

A patient (female, 52 years old) with the diagnosis of parapharyngeal space tumor (Figure 6A) was determined by fine needle aspiration to be a pleomorphic adenoma. The tumor was found to be extending to the lateral poststyloid space on CT imaging (Figure 6B). A 3D model for this patient was reconstructed and the tumor was detected to extend posterolateral to the styloid process (Figure 6C). The relationship between the tumor with the adjacent structures (i.e., ICA, external carotid artery, internal jugular vein, middle cranial fossa, mandibular, and maxillary) was demonstrated in detail (Figure 6D).

## 4 DISCUSSION

Structures including the pICA, internal jugular vein, and lower cranial nerves in the UPPS are accessible by EEA,^20^, ^21^, ^22^ whereas the styloid process is considered to be the boundary of the posterolateral extension within the ITF. Therefore, further extension into the lateral poststyloid space as described in the present study represents an “extension” of the EEA corridor.

This study confirms that exposure of the styloid process and its attached muscles through an endonasal corridor is difficult when the tympanic portion of the temporal bone is prominent,^16^ which was found in 5/12 sides (41.67%). In this setting, the exposure of structures including the internal jugular vein and lower cranial nerves within the poststyloid compartment at its posterior aspect was restricted. Removal of the styloid process and the tympanic portion of the temporal bone increases the space for maneuverability of the poststyloid space as well as control of the internal jugular vein, which may have implications for the management of lesions arising from the poststyloid UPPS compartment via an endoscopic endonasal or transoral approach.^23^ However, the procedure is technical demanding; and as such, its application still deserves critically clinical validation.

The jugular bulb is in continuity with the internal jugular vein when traveling downward into the UPPS.^24^ An open approach, such as an infratemporal fossa type A corridor, has been considered as the traditional method for management of lesions arising from the jugular foramen region (e.g., paraganglioma, meningioma, schwannoma).^25^, ^26^ However, few studies have addressed the maximal extent of exposure of the jugular bulb through an endoscopic approach. In this cadaveric study, only the anteroinferior aspect of the jugular bulb could be accessed through an endonasal approach, which might be suitable for managing lesions arising in the ITF with posterolateral extension toward the jugular bulb. However, the endoscopic approach is contraindicated for lesions located at the superior, lateral, and posterior aspects of the jugular bulb.^27^ Furthermore, control of the upper segment of internal jugular vein and the jugular bulb through a pure endoscopic approach was significantly hindered by decreased instrument maneuverability. An external approach seems better suited to control the internal jugular vein or the jugular bulb when encountering complex situations.

The dominating structure at the lateral poststyloid space include the main trunk of the extratemporal facial nerve, which exits the stylomastoid foramen to enter the parotid gland.^28^ In this study, exposure of the lateral poststyloid space provided an alternative to access the region lying between the styloid process and the facial nerve, which also represents an extreme lateral extension accessed through an endoscopic endonasal corridor. Appreciation of the anatomical course of the facial nerve through this corridor is beneficial for management of lesions arising from the UPPS with extension into this region, further safeguarding the facial nerve. Moreover, the accompanying vessel (stylomandibular artery) to the facial nerve is also prominent and not difficult to control through an endoscopic approach (e.g. harmonic scalpel or bipolar electrocautery). The accompanying vessel can also be controlled without difficulty through an external corridor when the facial nerve reanimation is necessitated at the level of stylomastoid foramen.^29^

Multiple branches arising from V_3_ innervate the muscles of mastication.^30^ In all 12 sides, we found that the deep temporal nerve, the motor nerve to the temporalis muscle, was constantly located along the medial border of the temporalis muscle. The deep temporal nerve was detected to have an identical diameter with the extratemporal facial nerve, which was accessed in the same surgical field through an endonasal approach; moreover, the transected deep temporal nerve could be adequately transposed to the stylomastoid foramen. Therefore, the deep temporal nerve is potentially a donor nerve for reanimation of a facial nerve paralysis (lesion above the level of stylomastoid foramen). This possibility requires additional investigation and would require a complementary open approach for the neural anastomosis.^31^

Selecting a surgical approach for management of lesions extending to the lateral poststyloid space mainly depends on the lesion location and dimension.^32^ For small to medium sized lesions (<4 cm) arising in the deep lobe of the parotid gland, accesses including the transcervical‐parotid, transoral and transnasal approaches can provide adequate exposure.^33^ For lesions with a diameter larger than 4 cm, however, endoscopic transnasal or transoral corridor may not guarantee en bloc extirpation, which is not suitable for pleomorphic adenoma; and as such, the traditional transcervical‐parotid approach is still the optimal strategy.^32^ For lesions arising from the nasal cavity, nasopharynx, pterygopalatine fossa, and the infratemporal fossa with an extension into the lateral poststyloid space; however, the transnasal or a combined transnasal with an open approach may be a good option.

The inferior head of the lateral pterygoid muscle was observed to be located immediately anterior to the UPPS.^4^ Resection of the inferior head of lateral pterygoid muscle was required to facilitate a far‐lateral extension of the lateral poststyloid space as described in the present study. Therefore, the superior head of the lateral pterygoid muscle and the medial pterygoid muscle could be preserved from a technical perspective, which might be beneficial for improving postoperative trismus. However, coping with the potentially significant bleeding from the pterygoid plexus and the effect of preserving the superior head of lateral pterygoid muscle on postoperative trismus need to be properly evaluated in the proper clinical setting. In addition, exposure of structures in the lateral poststyloid space including pICA, internal jugular vein and the facial nerve through endoscopic approach was within the endoscopic working distance based on our measurements (Table 1).

This study carries significant limitations. Although endoscopic endonasal accessing the lateral poststyloid space was feasible on a cadaveric model, one should note that this is a preclinical study and the application of this technique in live surgery still needs to be validated. Similarly, while the deep temporal nerve carries motor function, its actual outcome on facial nerve reanimation with the facial nerve requires clinical validation. Furthermore, copious bleeding from the deep pterygoid venous plexus when elevating the medial aspect of the lateral pterygoid muscle should be anticipated; and as such, sufficient preparation is critical before elevating the lateral pterygoid muscle. In addition, any endonasal attempt to address lesions arising in the lateral poststyloid space should be highly selective and only performed in centers with sufficient experience in endoscopic skull base surgery.

## 5 CONCLUSION

This study validated the feasibility to access the lateral poststyloid space via an endonasal approach. One could access the main trunk of the extratemporal facial nerve by removing the styloid process and its attachments; thus, providing the possibility to manage lesions deep to the facial nerve as well as reanimating the facial nerve with the deep temporal branch of V_3_.

## CONFLICT OF INTEREST

Nyall R. London holds stock in Navigen Pharmaceuticals, was a consultant for Cooltech Inc., and receives research funding from Merck, Inc., none of which are relevant to this study. The other authors have no funding, financial relationships, or conflicts of interest to disclose.

## Data Availability

The data that support the findings of this study are available from the corresponding author upon reasonable request.
